# Manipulation of the Size and Phase Composition of Yttrium Iron Garnet Nanoparticles by Pulsed Laser Post-Processing in Liquid

**DOI:** 10.3390/molecules25081869

**Published:** 2020-04-17

**Authors:** Tim Hupfeld, Frederic Stein, Stephan Barcikowski, Bilal Gökce, Ulf Wiedwald

**Affiliations:** 1 Technical Chemistry I and Center for Nanointegration Duisburg-Essen (CENIDE), University of Duisburg-Essen, Universitaetsstrasse 7, 45141 Essen, Germany; tim.hupfeld@uni-due.de (T.H.); frederic.stein@uni-due.de (F.S.); stephan.barcikowski@uni-due.de (S.B.); 2 Faculty of Physics and Center for Nanointegration Duisburg-Essen (CENIDE), University of Duisburg-Essen, Lotharstr. 1, 47057 Duisburg, Germany

**Keywords:** yttrium iron oxide, perovskite, garnet, phase transformation, ferrimagnetic nanoparticles, laser ablation, laser fragmentation, laser melting, monodisperse

## Abstract

Modification of the size and phase composition of magnetic oxide nanomaterials dispersed in liquids by laser synthesis and processing of colloids has high implications for applications in biomedicine, catalysis and for nanoparticle-polymer composites. Controlling these properties for ternary oxides, however, is challenging with typical additives like salts and ligands and can lead to unwanted byproducts and various phases. In our study, we demonstrate how additive-free pulsed laser post-processing (LPP) of colloidal yttrium iron oxide nanoparticles using high repetition rates and power at 355 nm laser wavelength can be used for phase transformation and phase purification of the garnet structure by variation of the laser fluence as well as the applied energy dose. Furthermore, LPP allows particle size modification between 5 nm (ps laser) and 20 nm (ns laser) and significant increase of the monodispersity. Resulting colloidal nanoparticles are investigated regarding their size, structure and temperature-dependent magnetic properties.

## 1. Introduction

Magnetic mixed metal oxide nanoparticles are an important class of materials for catalysis [1,2], biomedicine [3,4,5], and nanoparticle-polymer composites [6,7] and are also of high interest for applications in additive manufacturing, e.g., for 4D printing of magnetic structures [8,9,10]. For many of these applications, nanoparticles are required in colloidal form, dispersed in liquids such as pure water, organic solvents or polymer solutions. As a green method for synthesis and size modifications of colloidal nanoparticles, laser ablation in liquid (LAL) and laser post-processing (LPP) [11] have proven to be scalable [12,13] and versatile regarding nanoparticle composition [14,15,16,17,18,19,20,21,22,23] and choice of the liquid medium [24,25,26,27,28]. By LAL, metal and metal oxide nanoparticles have been successfully generated in relevant amounts, which is a key requirement for application. To increase the nanoparticle yield from oxide targets in LAL, unwanted byproducts in the form of microparticles should be minimized, which is achieved by using mechanically stable targets. In this way, challenging materials like ternary oxide nanoparticles [29,30] or doped nanoparticles [31,32] can be produced.

One of these challenging materials is Y_3_Fe_5_O_12_ (yttrium iron garnet, YIG), which is widely used as a material for microwave devices [33]. YIG is also known for its outstanding magneto-optical properties [34,35,36] and low spin-wave damping [37]. Furthermore, YIG nanoparticles synthesized by LPP were successfully used in laser-based additive manufacturing of steel powder [38]. As a competing phase to YIG, YFeO_3_ (yttrium iron perovskite, YIP) also features excellent magneto-optical properties [39]. It is a canted antiferromagnet with a very low magnetization of 0.2 Am^2^kg^−1^ and a high domain wall velocity [39]. However, there are only a few studies available on the magnetic properties of ultra-small YIG and YIP nanoparticles <10 nm. It is known that the surface-to-volume ratio affects the anisotropy constant, which makes ultra-small YIG particles particularly interesting [40]. Schmitz et al. demonstrated a LAL approach to obtain YIG nanoparticles, followed by LPP of the colloid via ns laser fragmentation in liquid (ns-LFL) for subsequent nanoparticle size reduction and ended up with unexpected high coercive and irreversibility fields at low temperatures [30]. The generated nanoparticles were much smaller than by wet chemical approaches [40], but considering their volume-weighted particle size distribution, still, a significant number of larger particles above 10 nm was present. The broad size distribution is a disadvantage which hinders the correlation of interesting magnetic properties and size and phase of the nanoparticles. Typically, size control during LAL and LPP can be achieved by variation of laser parameters and the choice of specific additives or saline solutions [41,42,43,44]. Variation of salinity works well with noble metals like gold, but in the case of less noble metals, molecular oxygen can oxidize the resulting nanoparticles. Moreover, in the case of oxides, stabilization with salts does not work and macromolecular ligands can affect the chemical composition of the nanoparticles [29,30], which makes size control of oxide nanoparticles challenging.

To overcome this, we investigate LPP of ligand-free YIG colloids with ps and ns pulses to manipulate the particle size distribution and possibly the phase composition to get deeper insights into the magnetic properties of the resulting nanoparticles. 

## 2. Results and Discussion

### 2.1. Nanoparticle Size Modification

**Figure 1:**
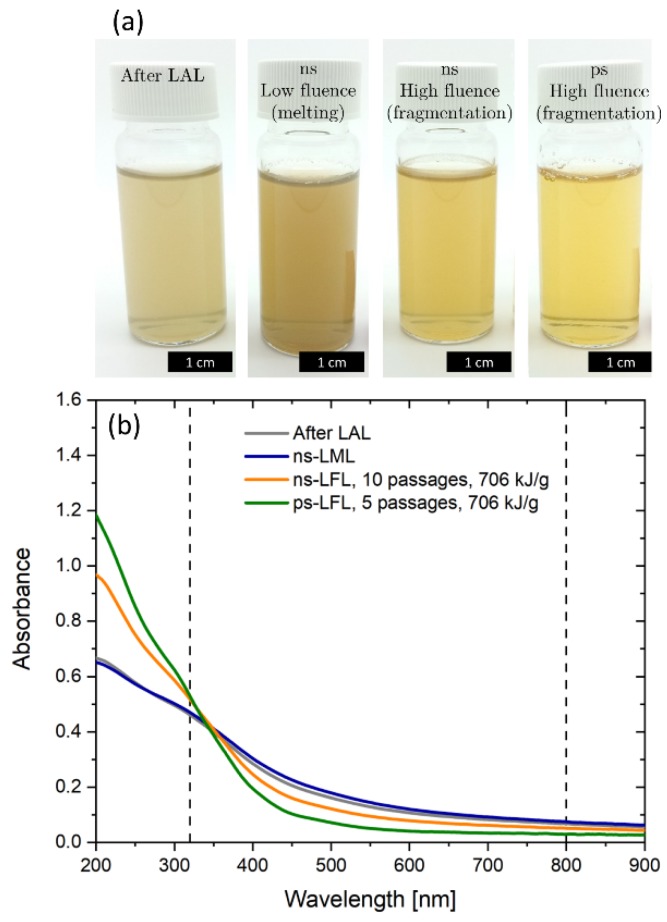
(**a**) Image of the educt colloid gained by laser ablation in water and the colloids after laser post-processing with different pulse durations and fluences at the same nanoparticle concentration. From left to right: educt, ns low fluence, ns high fluence, ps high fluence. (**b**) Corresponding UV-Vis-extinction spectra. Differences in the ratio of the absorbance at 320 and 800 nm (wavelength marked in the graph) already indicate differences in nanoparticle size distribution. An alternative measure for the process efficiency is the Furlong slope, presented in the Supplementary Material, Figure S3.

To achieve a particle size modification, the applied laser fluence is a crucial parameter. According to Lau et al. there are four different fluence regimes [45]. In the first regime, there is no effect of the laser irradiation on nanoparticle size (untreated, UT), in the melting regime, particles partially melt and/or fuse together (LML), in the fragmentation regime the fluence is large enough to fragmentize particles (LFL) and in the optical breakdown regime (OB), losses through ionization of the liquid reduce fragmentation efficiency. Figure 1a shows colloid samples right after LAL and after LPP with different fluences and pulse durations. As expected, the influence of laser fluence variation can be observed right from the clouding (flocculation) of the YIG colloids, since particle size directly influences the scattering intensity. Colloids show the most pronounced clouding after LAL and low fluence ns post-processing. After high fluence post-processing, the clouding is significantly reduced, indicating smaller particles. The lowest scattering intensity can be observed for high fluence ps-LFL. To quantify this effect, the ratio of the absorbance at 320 and 800 nm was calculated from UV-Vis absorbance spectra (Figure 1b). At 320 nm, YIG shows a concentration-dependent absorbance, whereas the scattering of larger particles dominates absorbance at 800 nm. This ratio gives a good impression on the scattering intensity and is expected to correlate with nanoparticle size, similar to the primary particle index (PPI) known for gold and ZnO colloids [45,46,47]. On this basis, one can calculate a process efficiency which is given by the Abs_320_/Abs_800_-ratio after post-processing relative to the Abs_320_/Abs_800_-ratio before post-processing (after LAL). 

**Figure 2:**
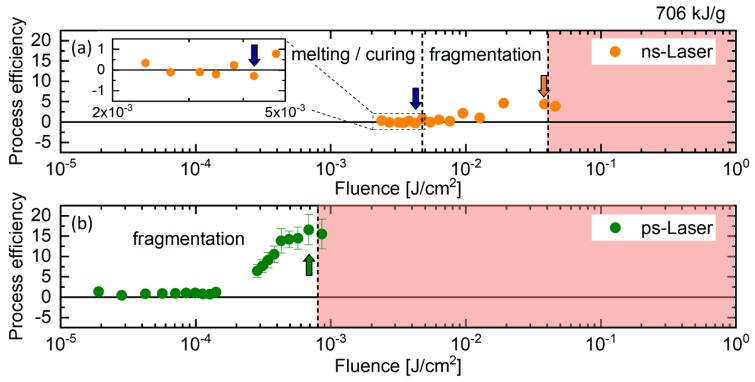
Process efficiency as a function of laser fluence for (**a**) ns and (**b**) ps post-processing. The process efficiency is based on the UV-Vis absorbance spectra and is calculated from the Abs_320_/Abs_800_-ratio after post-processing relative to the Abs_320_/Abs_800_-ratio before post-processing. The arrows indicate the optimum parameters for melting/curing (**blue**) and fragmentation (**orange** and **green**). In all cases, the specific energy input was 706 kJ/g. Note that the choice of specific energy dose represents a compromise between maximum fragmentation and minimum process duration. Further information on the variation of the specific energy dose can be found in Supplementary Materials, Figure S2.

The process efficiency as a function of fluence is depicted in Figure 2. For ns-LPP (Figure 2a), all the regimes mentioned above are passed as a function of fluence. Below 5 mJ/cm^2^, the process efficiency is negative, which indicates more scattering and slightly larger particles. There is some uncertainty regarding the transition between UT and LML regime since the absolute values of the process efficiency are rather small. Since we aimed for working in the LML and LFL regime, no further investigation of the transition between the UT and the LML regime was performed. Above 5 mJ/cm^2^ the process efficiency shows positive values (LFL regime) with a maximum at 30 to 40 mJ/cm^2^. As expected, the process efficiency does not increase further for higher fluences (OB regime). A similar trend can be observed for ps post-processing (Figure 2b), but the optical breakdown occurs at lower fluences due to the higher pulse energy of ps pulses. Note that deviations of the optical breakdown threshold from literature might be due to self-focusing effects of the cylindrical liquid jet, which results in a much higher fluence [48]. Compared to the ns-LFL, ps-LFL shows an approximately 200% higher process efficiency at the same specific energy input, which is attributed to the shorter pulse duration and less thermal energy losses through electron-phonon coupling and a higher pulse intensity due to shorter pulses. 

In contrast to ns irradiation, ps irradiation does not show any negative values for YIG in the investigated fluence range above 1.8 × 10^−5^ J/cm^2^ (no LML regime). In general, melting and fragmentation processes strongly depend on the pulse duration, the laser fluence, the particle absorption cross-section, and the thermal diffusion length in combination with the nanoparticle size or volume. For long pulse durations in the range of ns, more homogeneous heating can be expected and LML was observed in many studies. If the pulse duration is much shorter (e.g., ps pulses), homogeneous heating is unlikely and evaporation at the particle surface leads to nanoparticle byproducts even below the fragmentation threshold. To our best knowledge, there are just a few studies reporting ps-LML [45,49,50,51]. Sakaki et al. reported on burst-mode laser irradiation for homogeneous heating depending on the number of pulses and the interval between them to generate submicron spheres [50]. In other studies, high nanoparticle concentrations in the range of g/L were used for ps-LML [45,51], which is much higher than in our study and can lead to stability issues. All in all, we conclude that the chosen laser parameter, especially the laser fluence, in combination with the YIG colloid of the given concentration and particle size distribution, were not suitable for efficient ps-LML.

**Figure 3:**
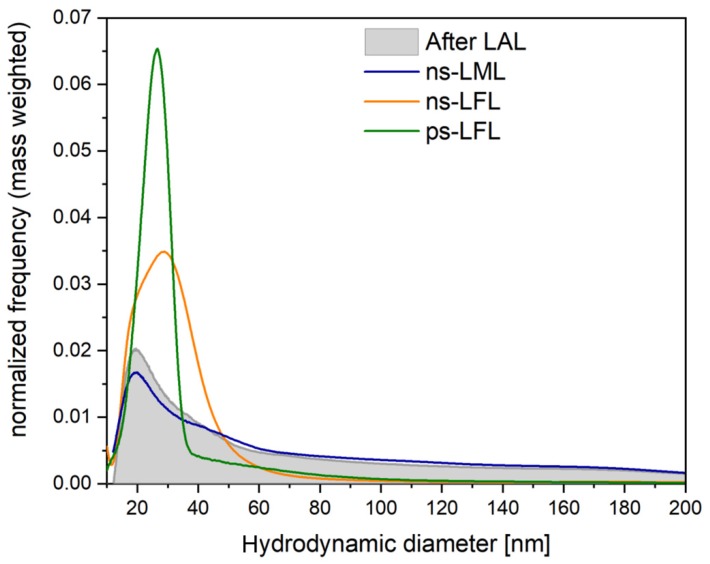
Mass-weighted hydrodynamic size distribution of the educt colloid after LAL and of the colloids after post-processing with lasers of different pulse durations and fluences, respectively.

Results of optical characterization and determination of the process efficiency are reflected in the mass-weighted hydrodynamic size distribution shown in Figure 3. In general, LFL results in a narrowed distribution at smaller particle size, whereby ps-LFL is much more efficient than ns-LFL. In contrast, ns-LML results in a preservation of the initial particle size distribution after LAL and only minor reduction of the particle´s mass fraction below 40 nm. Compared to ns-LFL, ns-LML features a slightly smaller peak maximum, but a broader distribution and a higher number of particles >60 nm. 

**Figure 4:**
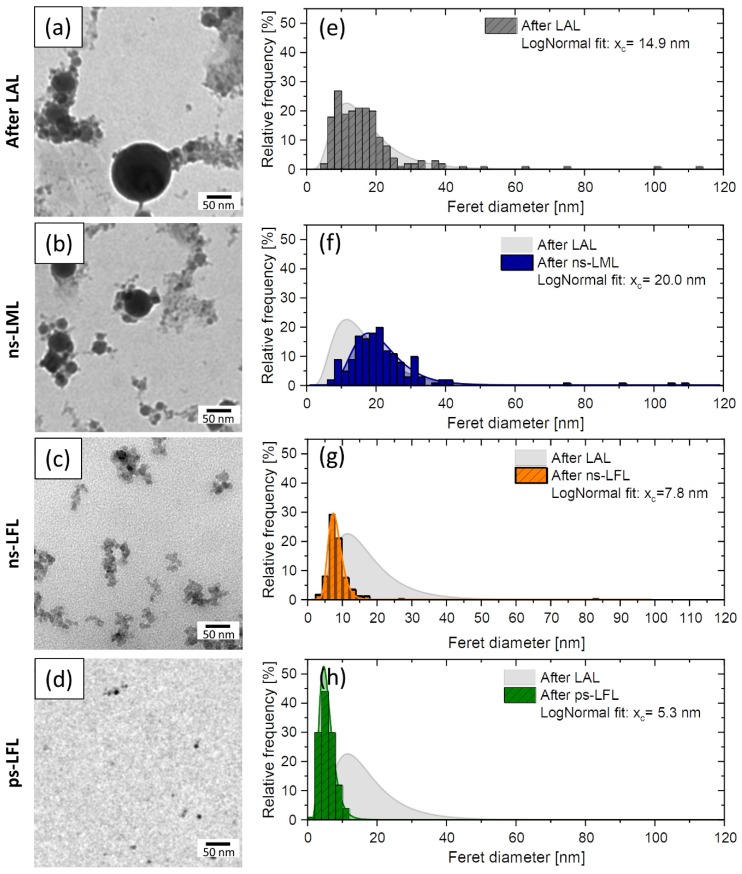
(**a**–**d**) TEM images and (**e**–**h**) corresponding size distributions (number-weighted) for the educt colloid after LAL and of the colloids after post-processing with lasers of different pulse durations and fluences. For each size distribution >500 particles were counted. The x_c_ value (expected value of the lognormal fit) is given for each size distribution.

TEM images in Figure 4 support these trends. Feret diameter distributions after LML show a clear difference between the educt and the LML-treated colloid. The xc-value (expected value) of the lognormal fit increases from 14.9 nm to 20.0 nm and the polydispersity index (PDI), which is calculated from the square of the expected value x_c_^2^ divided by its variance σ^2^, decreases from 0.33 to 0.14. This indicates an improved degree of monodispersity and an improved degree of monodispersity. LFL, on the other hand, significantly reduces the number of large particles >20 nm and leads to an x_c_-value of 7.8 nm for ns-LFL and 5.3 nm for ps-LFL, respectively. Monodispersity increases significantly from LAL generated colloids (PDI = 0.33) to ns-LFL (PDI = 0.17) and ps-LFL (PDI = 0.07). Overall, the size analysis clearly shows a trend toward ps-LFL being much more efficient than ns-LFL at the same specific energy input, leading to a narrower particle size distribution. Thus, we only compare ps-LFL, ns-LML with the educt after LAL in the following structural and magnetically characterization.

### 2.2. Structural Analysis

**Figure 5:**
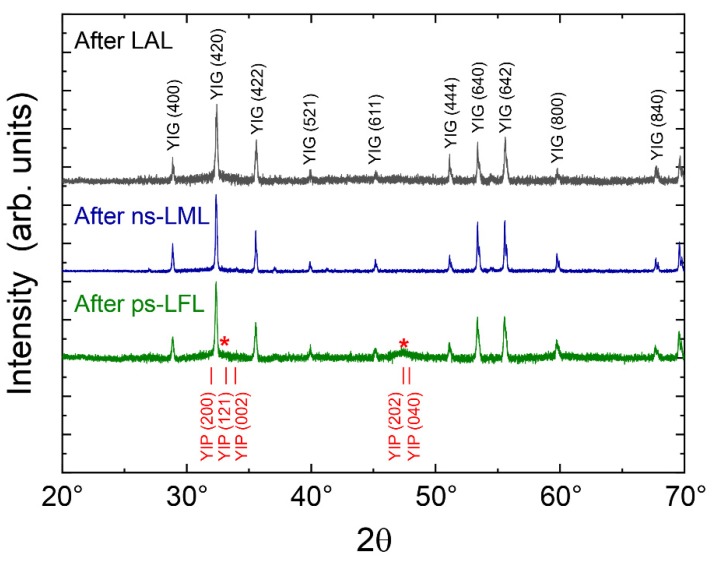
X-ray diffraction data after laser ablation in liquids (LAL), ns laser melting in liquids (ns-LML) and after ps laser fragmentation in liquids (ps-LFL). The pronounced YIG diffraction peaks (JCPDS PDF card 33-693) are indexed in black. Red stars indicate additional broad XRD features after ps-LFL. The positions of main diffraction peaks of YIP (JCPDS PDF card 39-1489.) are indexed in red (a plot with a logarithmic scale can be found in the Supplementary Materials, Figure S4).

X-ray powder diffraction is applied for the phase identification of the generated nanoparticles. Figure 5 presents the diffractograms on a linear scale after LAL, ns-LML, and ps-LFL. It is obvious that all diffractograms show the typical signature of YIG nanoparticles [52,53,54]. After LAL, however, an additional broad peak is found underneath the YIG(420) diffraction peak, which may indicate small grains of YIG or an additional phase. Note that the YIG(420) peak is the most prominent in powder XRD at about 32°. After ns-LML, the broad feature under the (420) peak vanishes and the YIG peaks further sharpen. This observation points to a growing grain size after the particles’ melting and is entirely in line with the observed particle growth in Figure 5. When ps-LFL is applied, the TEM size distribution gives a reduced particle size (x_c_ = 5.3 nm) and a sharper distribution. Although in the TEM investigations no larger particles have been found, it is clear from the diffractogram of the ps-LFL sample that the fragmentation is incomplete, and thus, some large YIG particles remain. Furthermore, the diffractogram exhibits again the broad feature overlapping with the YIG(420) diffraction peak and an additional broad peak at about 47° as indicated by the red stars. We carefully checked possible side phases such as several Fe oxides and Y_2_O_3_, since phase transformation can occur for LPP of oxide colloids [55], but none of those fits with their largest diffraction peaks to the two broad features (red stars). Another Y-Fe oxide, namely the yttrium iron perovskite phase YFeO_3_ (YIP), exhibits several diffraction peaks in the respective range, as reported by Nagrare et al. for YFeO_3_ nanocrystals [56]. Considering the small crystallite size of about 5 nm, several closely located XRD peaks will overlap and merge. As a result, very broad diffraction peaks appear and only a few, well-separated diffraction ‘bands’ can be distinguished. We conclude that it is likely that YFeO_3_-like nanocrystallites, presumably highly distorted, form by ps-LFL. 

Further evaluation of the XRD data yields the crystallite size by applying the Scherrer equation on the (420) peak at about 32°. We obtain crystallite sizes of 30 ± 5 nm, 48 ± 8 nm, and 35 ± 6 nm after LAL, ns-LML, and ps-LFL, respectively, and conclude that ns-LML enhances the crystallite size while the XRD crystallite size is much larger than the most probable TEM diameter. This arises from the small number of large particles comprising a dominant scattering volume in XRD. However, the tendency of an increased size by ns-LML is clear from both TEM and XRD investigations. Fragmentation, on the other side has no significant influence on the XRD grain size of the YIG phase. We ascribe this to a very small, remaining fraction of non-fragmented educt particles in the ps-LFL processed colloid. The additional peaks indicated by the red stars correspond to a crystallite size of 4.5 ± 1.5 nm. This value is in good agreement with the size distribution after ps-LFL. 

In summary, the structural investigations suggest the formation of larger YIG crystallite sizes after ns-LML, while ps-LFL leads to smaller particles, crystallized probably in the YIP phase. A further magnetic inspection may allow identifying these two distinct phases. 

### 2.3. Magnetic Properties

**Figure 6:**
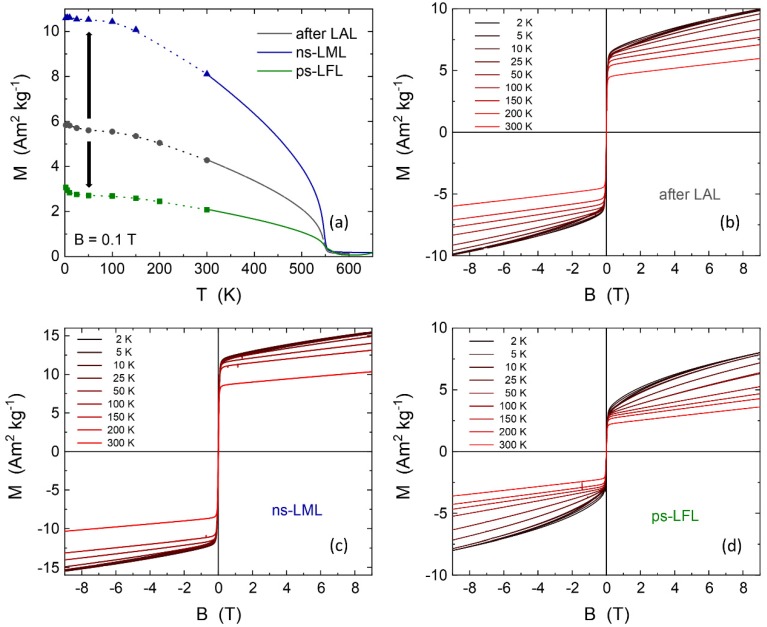
Magnetic properties of laser-generated yttrium iron oxide nanoparticles: (**a**) Magnetization as a function of temperature in B = 0.1 T after LAL, ns-LML, and ps-LFL. Points below 300 K are extracted from the hysteresis loops after LAL (**b**), ns-LML (**c**), and ps-LFL (**d**) connected by the dotted lines as guides to the eye. Data above 300 K were continuously recorded.

The magnetic properties of the educt and further sample processing can help to identify both the phase and the size of magnetic nanoparticles. Starting with the sample after LAL, it can be expected to find different magnetic properties after ns-LML and ps-LFL. Figure 6a presents the magnetization of the three samples as a function of temperature. The points below 300 K were extracted from the magnetic hysteresis loops after LAL, ns-LML, and ps-LFL, respectively (Figure 6b–d). The dotted line is a guide to the eye. Data above 300 K were continuously recorded in the vibrating sample magnetometer. All samples show the expected behavior for ferrimagnetic YIG with a Néel temperature of about 550 K. This is essentially the Néel temperature of YIG single crystals of *T*_N_ = 553 K [39] (and references in there) reflecting the high-quality YIG produced by LAL and the post-processing steps. The absolute values of the magnetization after LAL of 6 Am^2^kg^−1^ at low temperatures is, however, strongly reduced as compared to a single crystal (26.8 Am^2^kg^−1^) [39] or the lower magnetization of YIG nanoparticles of similar size (*M*_S_ = 10 Am^2^kg^−1^ for a diameter of 14 nm) [40]. Interestingly, the magnetization changes upon post-processing. When ns-LML is applied, we obtain *M* = 10.5 Am^2^kg^−1^ confirming the above *M*_S_ of similar-sized particles [40]. Thus, ns-LML improves the nanocrystal quality and may transform quasi-amorphous particles and YIP phases to YIG nanocrystals.

When ps-LFL is applied, the magnetization decreases to about 3 Am^2^kg^−1^ at low temperatures, where additionally a slight hyperbolic decrease appears. The latter feature can be ascribed to paramagnetic species generated by ps-LFL. Nonetheless, the major contribution to the temperature-dependent magnetization still shows the YIG volume Néel temperature. The magnification of the high-temperature region (T > 400 K, zoom-in is presented in Supplementary Materials, Figure S5), however, gives a clear indication of a second magnetic species with higher ordering temperature. We suggest that *T*_N2_ = 570 K arising after ps-LFL is due to the formation of the YIP phase from larger YIG nanoparticles. YIP is a canted antiferromagnet. Single crystals of YIP have a Néel temperature of 643 K and a very low magnetization of 0.2 Am^2^kg^−1^ [39]. It is often found that for small particles the magnetic ordering temperature is reduced. Due to the low magnetization of the canted antiferromagnet, we can expect that YIP only shows up in magnetometry when the relative amount is rather large. Assuming the full magnetization of YIG nanoparticles develops in all samples (*M*_YIG_ = 10.5 Am^2^kg^−1^), the reduction to 3 Am^2^kg^−1^ for ps-LFL is equivalent to a phase composition of about 30% YIG and 70% of a second, quasi-antiferromagnetic phase (presumably YIP) after ps-LFL. Note that magnetometry measures the mass averaged magnetization. 

Further analysis of the magnetic hysteresis loops at various temperatures in Figure 6b–d also exhibits the features of the two phases. After LAL, a very soft magnetic hysteresis loop is observed as expected for YIG. After saturation of this component well below 1 T, a paramagnetic slope is recorded. This can arise from a (quasi-)antiferromagnetic phase, presumably the canted antiferromagnetic YIP phase, as it has been observed before for 30 nm and 60 nm YFeO_3_ nanoparticles. Prokov et al. reported a high field susceptibility of χ_HF_ = 0.004 emu mol^−1^Oe^−1^ at *T* = 4 K and slightly lower at 300 K [57]. For comparison, we calculated the high field susceptibility in the identical units. The present samples show χ_HF_ = 0.003 and 0.006 emu mol^−1^Oe^−1^ for 300 K and 5 K, respectively, which is comparable to earlier results for pure YFeO_3_. Such a relatively small variation of χ_HF_ by only 50% from 5–300 K is inconsistent with a Langevin paramagnet which shows a hyperbolic decrease. Thus, we have three parameters, i.e., the reduced magnetization, the second Néel temperature *T*_N2_, and the high field susceptibility, suggesting the formation of the second (main) phase at about 70% phase content after ps-LFL. All these features and in the light of the structural investigations, it is likely that a highly distorted YIP-like phase forms.

## 3. Materials and Methods

Iron oxide (Fe_2_O_3_) and yttrium oxide (Y_2_O_3_) nanopowders for target manufacturing were both purchased from Sigma-Aldrich (St. Louis, MO, USA), homogeneously mixed and pressed at 330 MPa. Thereafter, the green compacts were sintered at 1550 °C for 6 h (Nabertherm LHT 01/17D, Lilienthal, Germany) to create targets with a pure YIG phase (details on Target manufacturing are shown in [30]). Since there is an influence of the target porosity on the nanoparticle yield, only dense targets with a density of more than 95% compared to the bulk density were used for laser ablation in liquids (LAL). 

**Figure 7:**
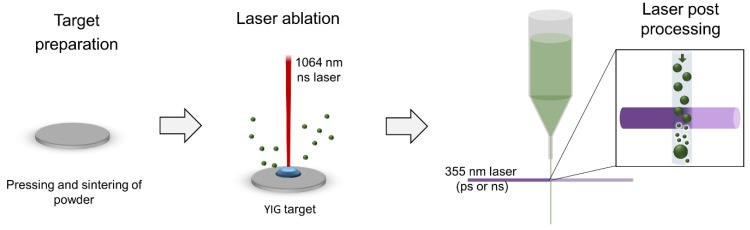
Schematic illustration of process steps for laser-synthesis of ligand free nanoparticles from powder materials. From left to right: target manufacturing, laser ablation in liquid (LAL) and laser post processing (LPP) of colloids.

LAL was performed in pure water, obtained from a Milli-Q purification system (Merck, Darmstadt, Germany), with a 1064 nm Nd:YAG-laser in a batch setup introduced in [58]. At 10 kHz, the laser (Rofin PowerLine E, Hamburg, Germany) delivered 8 ns pulses with a fluence of 16.7 J/cm^2^, and LAL was performed for 5 min. Analog to earlier studies [30,45,59], a liquid jet setup was used for laser post-processing (LPP) of the laser-generated YIG colloids with a concentration of 160 mg/L (Figure 7). The colloid was directed through a glass nozzle (60 mL/min), forming a liquid jet with a diameter of 1.3 mm, which was irradiated with either ns- or a ps-pulses at 80 kHz repetition rate (Coherent Avia 355-23, Santa Clara, CA, USA, or Edgewave PX400-3-GH, Würselen, Germany). Since YIG is well absorbing in the UV range below a wavelength of 370 nm, the third harmonic of the lasers was used by calculation of absorption efficiency and melting threshold (see Supplementary Materials, Figure S1. for details on wavelength selection). The liquid jet reactor allows flexible tuning of the applied fluences, just by changing the distance between the lens and the liquid jet. Further, it is possible to irradiate colloids several times (several passages) to increase the specific energy dose. According to Lau et al. the specific energy dose is calculated by subtracting the transmitted laser powder from the nominal laser power without liquid jet and normalization of this value to the nanoparticle concentration and volume flow rate [45]. In the present case, roughly 10–20% of the laser power was transmitted through the liquid jet to the power meter and showed fluctuations of several %. Furthermore, scattering effects caused by the nanoparticles and diffraction effects at the air-water-interface of the liquid jet make the calculation of the dose rather inaccurate [48]. Therefore, we ignore transmission and calculate the nominal specific energy input right from the laser output power in front of the liquid jet and normalized to the nanoparticle concentration and volume flow rate.

After LAL and LPP, absorbance spectra in the UV-Vis range were collected for all colloids (Thermo Scientific Evolution 201, Waltham, MA, USA) and hydrodynamic size distribution was measured by analytical disc centrifugation (ADC, CPS Instruments, Prairieville, LA, United States). Transmission electron microscopy (TEM, Zeiss EM 910, Oberkochen, Germany) and X-ray powder diffraction (XRD, PANalytical X’Pert PRO, Almelo, Netherlands) were used for more information on the Feret particle size and the phase composition. Finally, the magnetic properties of dried powders were characterized in a vibrating sample magnetometer (VSM, Quantum Design MPMS XL, Darmstadt, Germany).

## 4. Conclusions

Laser post-processing (LPP) of colloids is a powerful tool for the modification of particle size and phase composition of magnetic mixed oxide nanoparticles and therefore an important aspect for their applicability. Irradiating a laser-generated YIG colloid with ps laser pulses (ps-LFL) results in particle fragmentation from 14 to 5 nm, accompanied by a significant increase of monodispersity. ps-LFL is found to be much more efficient for fragmentation as compared to ns-LFL at the same specific energy dose. At low fluences, however, ns-irradiation allows laser melting (ns-LML) and an increase of particle size to 20 nm. Furthermore, purification of the YIG phase occurs during ns-LML, which is reflected by an increase of magnetization from 6 Am^2^kg^−1^ to 10.5 Am^2^kg^−1^ in B = 0.1 T, in accordance to values measured for similar-sized YIG particles in literature. In turn, ps-irradiation reduces the magnetization to 3 Am^2^kg^−1^ and gives a clear indication of a second magnetic species with a higher Néel temperature than YIG. Considering the two Néel temperatures after ps-LFL, the low magnetization which has been increased by ns-LML and decreased by ps-LFL, and a high field susceptibility, it is very likely that ps-LFL transforms YIG nanoparticles to another nanocrystalline species with small particle size. From the measured magnetization, we were also able to approximate a phase composition of 30% YIG and 70% of a second (quasi-)antiferromagnetic phase, presumably Yttrium Iron Perovskite (YIP). Our results underline the flexibility of LPP for modification of oxide nanomaterials, which could enable better applicability, e.g., in nanoparticle-polymer composites.

## Supplementary Materials

The following are available online Figure S1: (a) Absorption efficiency Q_abs^λ as a function of the particle size (b) Estimation of the fluence required for melting particles (yellow line) and evaporate them (gray line); Figure S2: UV-Vis absorbance ratio between 320 and 800 nm as a function of the (a) number of passages during fragmentation and (b) specific energy input in kJ/g which considers, that the ps-laser has twice the pulse energy and total laser power compared to the ns-laser; Figure S3: (a) Double logarithmic plot of the UV-Vis absorbance spectra to calculate (b) the Furlong slope from the linear slope between 250 and 300 nm; Figure S4: Experimental data of the ps-LFL sample in logarithmic scale together with the YIP reference positions and intensities (JCPDS PDF card 39-1489); Figure S5: Magnetization as a function of temperature at B = 0.1 T in the high-temperature region after ps-LFL. Two ordering temperatures TN1 and TN2 are detected.
