# Supplementary material for: Manipulation of the Size and Phase Composition of Yttrium Iron Garnet Nanoparticles by Pulsed Laser Post-Processing in Liquid
Source: Molecules. 2020 Apr 17;25(8):1869. doi: 10.3390/molecules25081869 (PMC7221795; doi:10.3390/molecules25081869)
Supplement: Supplementary file 1 [file molecules-25-01869-s001.pdf]

Supporting information

Manipulation of the size and phase composition of yttrium iron garnet nanoparticles by laser post-processing in liquid

Tim Hupfeld[1], Frederic Stein[1], Stephan Barcikowski[1], Bilal Gökce[1], and Ulf Wiedwald[2]

[1] Technical Chemistry I and Center for Nanointegration Duisburg-Essen (CENIDE), University of Duisburg-Essen, Universitaetsstrasse 7, 45141 Essen, Germany
[2] Faculty of Physics and Center for Nanointegration Duisburg-Essen (CENIDE), University Duisburg-Essen, Lotharstr. 1, 47057 Duisburg, Germany

*corresponding authors: bilal.goekce@uni-due.de, ulf.wiedwald@uni-due.de

1. Choice of laser wavelength for LPP

The energy that can be absorbed by a particle is strongly reliant on its wavelength-dependent absorption cross-section σ_{abs}^{λ} [1].

$$E_{abs} = \frac{E_P}{S_P} \cdot \sigma_{abs}^{\lambda} = J_P \cdot \sigma_{abs}^{\lambda} \tag{1}$$

For spherical particles, a relative absorption cross-section Q_{abs}^{λ}, also known as absorption efficiency, can be calculated with the geometrical cross-section $\frac{\pi \cdot d_P^2}{4}$:

$$Q_{abs}^{\lambda} = \frac{4 \cdot \sigma_{abs}^{\lambda}}{\pi \cdot d_P^2} \tag{2}$$

The size- and wavelength-dependent absorption cross-section can be determined by calculating the absorption efficiency using the classical Mie theory. It describes the interaction between a planar electromagnetic wave and a homogeneous sphere [2]. To calculate the absorption efficiency, it is necessary to know the real and imaginary part of the complex refractive index of the material at the respective laser wavelengths. Kahn et al. determined the values experimentally for the complex refractive index of YIG, which are used as a basis for the calculation in this paper [3]. Although there is no general limit for the particle size to be calculated in Mie theory, it should be noted that quantum mechanical effects can play an essential role in particles below 10 nm and must, therefore, be considered. For the sake of simplicity, the smaller particle size is thus set to 10 nm. Figure S1 shows the absorption curves for YIG at three usual laser wavelengths of 1064 nm, 533 nm, and 355 nm. Larger wavelengths can only be efficiently absorbed by large particles, whereas the absorption efficiency for particles below 1000 nm decreases rapidly for 1064 nm. By reducing the wavelength to 532 nm, it is possible that particles down to 300 nm can absorb the energy efficiently. For an efficient fragmentation

process, however, it is essential that large amounts of energy can also be absorbed by particles smaller than 100 nm, which is why a laser with a wavelength of 355 nm is best suited for our study. Using the model of Takami et al.'s "heating melting evaporation" theory, the amount of energy absorbed by a YIG particle of any size can now be calculated [4]. This work has not succeeded in determining the specific enthalpies at each temperature required for the calculations, which is why the curves in (b) provide only trends and no exact values. From the application, it can be concluded that significantly more energy is required for the evaporation and thus the fragmentation of small particles below 100 nm than for larger particles. This confirms the conclusion that the use of a 355 nm wavelength laser is the optimal choice for the fragmentation of small YIG particles.

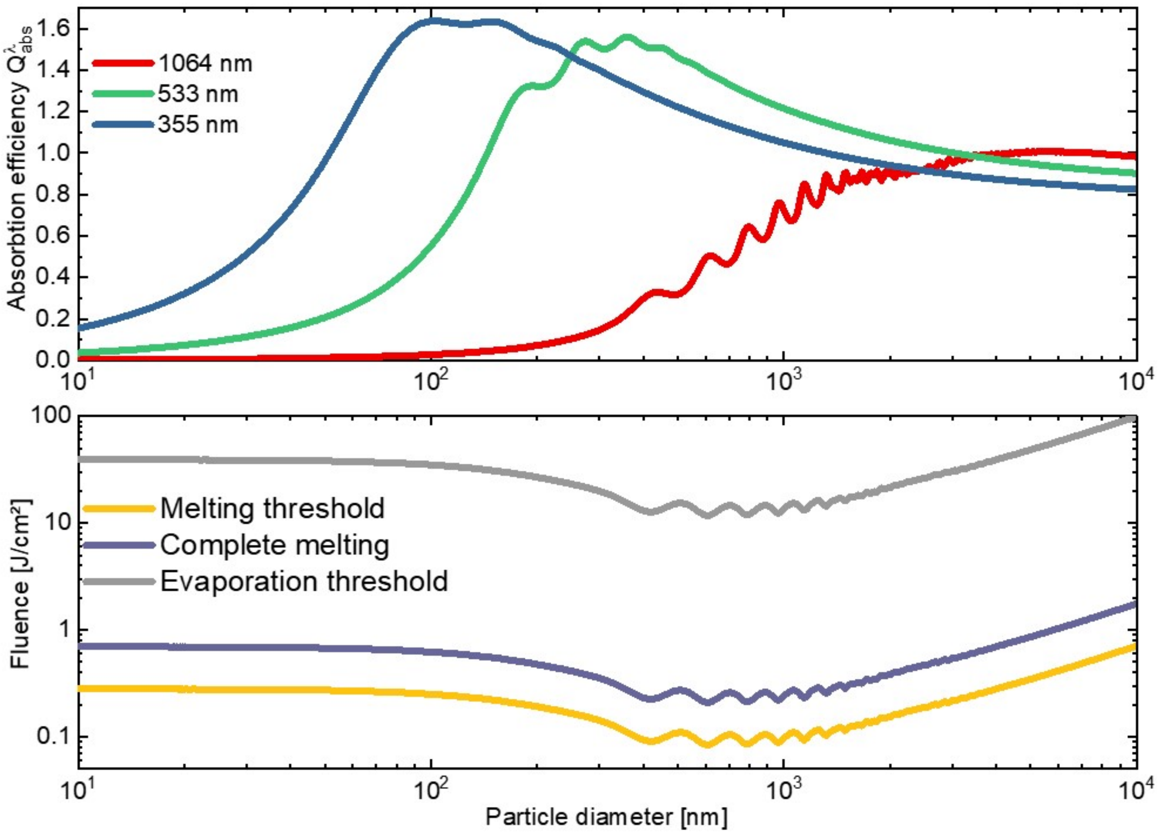

Figure S1: (a) Absorption efficiency Q_{abs}^{λ} as a function of the particle size, which was calculated using the Mie theory. (b) Estimation of the fluence required for melting particles (yellow line) and evaporate them (gray line), calculated using the "heating-melting-evaporation" model according to Takami et al. [4].

2. Influence of specific energy input on the UV-Vis absorbance ratio between 320 and 800 nm

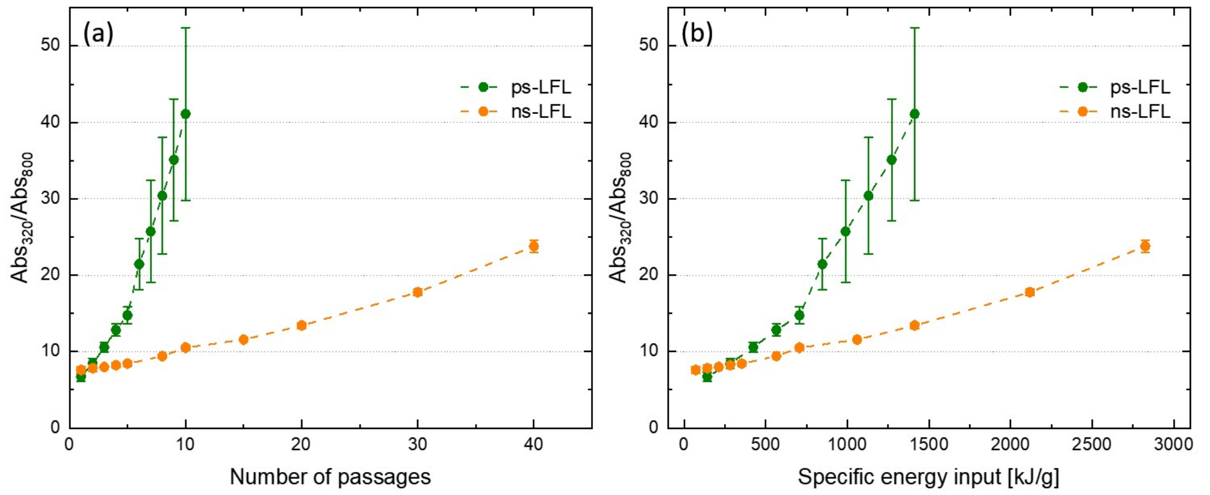

Figure S2: UV-Vis absorbance ratio between 320 and 800 nm as a function of the (a) number of passages during fragmentation and (b) specific energy input in kJ/g which considers, that the ps-laser has twice the pulse energy and total laser power compared to the ns-laser.

3. Furlong slope

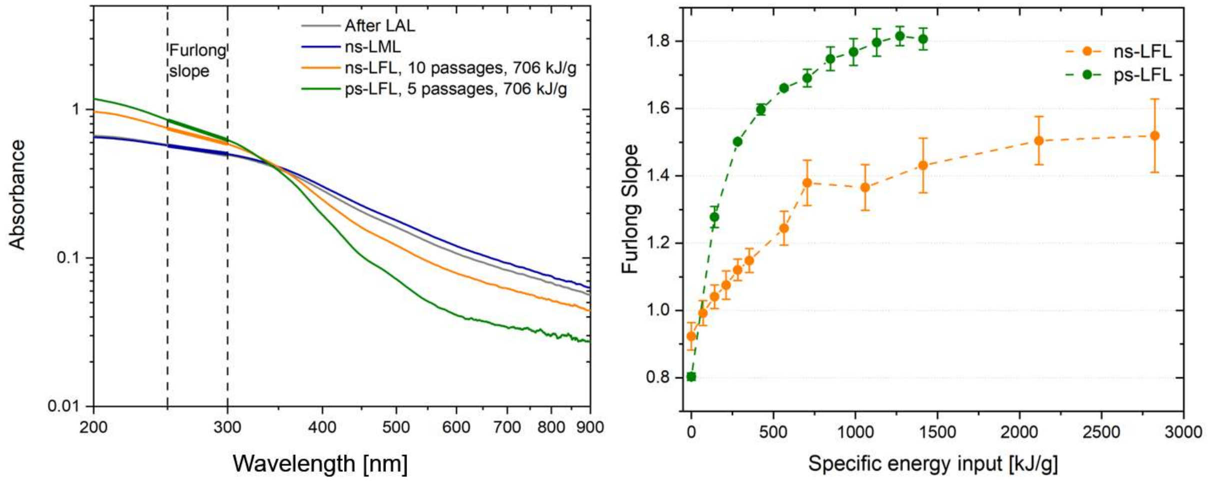

Figure S3: (a) Double logarithmic plot of the UV-Vis absorbance spectra to calculate (b) the Furlong slope from the linear slope between 250 and 300 nm.

4. XRD results after ps-LFL

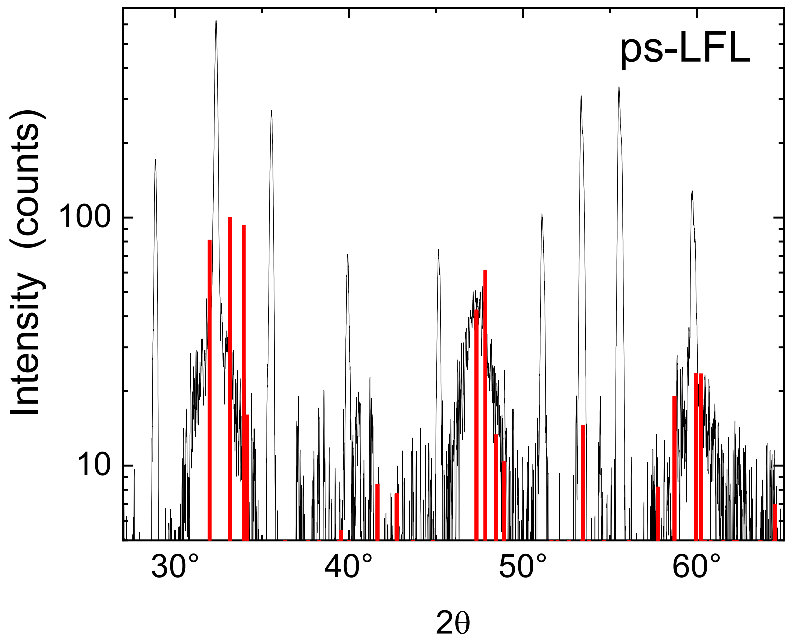

Figure S4: Experimental data of the ps-LFL sample in logarithmic scale together with the YIP reference positions and intensities (JCPDS PDF card 39-1489).

Figure S4 shows that the angular position of the broad features under discussion (32°, 47°, and 60°) are slightly shifted towards lower angles with respect to the reference data. This might be due to a distorted lattice. Note that the grain size is very small (TEM and XRD data) and such variations are possible. Nonetheless, the main features (considering the overlap of several diffraction planes to one peak observed in the experimental data) are consistent with the phase group 62 (Pnma), so presumably YIP.

5. M(T) for high temperatures

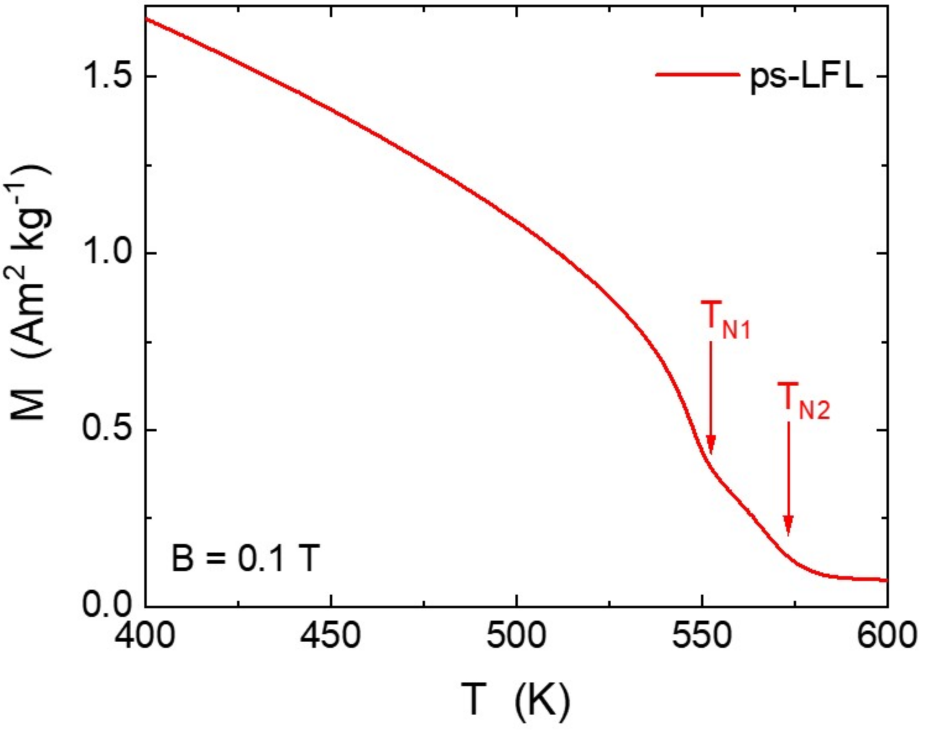

Figure S5: Magnetization as a function of temperature at B = 0.1 T in the high-temperature region after ps-LFL. Two ordering temperatures T_{N1} and T_{N2} are detected.

References

[1] A. Pyatenko, M. Yamaguchi, M. Suzuki, Mechanisms of size reduction of colloidal silver and gold nanoparticles irradiated by Nd:YAG laser, J. Phys. Chem. C. 113 (2009) 9078–9085. doi:10.1021/jp808300q.

[2] G. Mie, Beiträge zur Optik trüber Medien, speziell kolloidaler Metallösungen, Ann. Phys. 25 (1908) 377.

[3] F.J. Kahn, P.S. Pershan, J.P. Remeika, Ultraviolet magneto-optical properties of single-crystal orthoferrites, garnets, and other ferric oxide compounds, Phys. Rev. 186 (1969) 891–918. doi:10.1103/PhysRev.186.891.

[4] A. Takami, H. Kurita, S. Koda, Laser-induced size reduction of noble metal particles, J. Phys. Chem. B. 103 (1999) 1226–1232. doi:10.1021/jp983503o.
